# Fluorescent-Based Neurotransmitter Sensors: Present and Future Perspectives

**DOI:** 10.3390/bios13121008

**Published:** 2023-11-30

**Authors:** Rajapriya Govindaraju, Saravanan Govindaraju, Kyusik Yun, Jongsung Kim

**Affiliations:** 1 Department of Chemical and Biological Engineering, Gachon University, 1342 Seongnam Daero, Seongnam-si 13120, Gyeonggi-do, Republic of Korea; mhbrenga@gmail.com; 2 Department of Bio Nanotechnology, Gachon University, Seongnam-si 13120, Gyeonggi-do, Republic of Korea; biovijaysaran@gmail.com (S.G.); ykyusik@gachon.ac.kr (K.Y.)

**Keywords:** neurotransmitters, biosensing, fluorescence, nanomaterials, biomaterials

## Abstract

Neurotransmitters (NTs) are endogenous low-molecular-weight chemical compounds that transmit synaptic signals in the central nervous system. These NTs play a crucial role in facilitating signal communication, motor control, and processes related to memory and learning. Abnormalities in the levels of NTs lead to chronic mental health disorders and heart diseases. Therefore, detecting imbalances in the levels of NTs is important for diagnosing early stages of diseases associated with NTs. Sensing technologies detect NTs rapidly, specifically, and selectively, overcoming the limitations of conventional diagnostic methods. In this review, we focus on the fluorescence-based biosensors that use nanomaterials such as metal clusters, carbon dots, and quantum dots. Additionally, we review biomaterial-based, including aptamer- and enzyme-based, and genetically encoded biosensors. Furthermore, we elaborate on the fluorescence mechanisms, including fluorescence resonance energy transfer, photon-induced electron transfer, intramolecular charge transfer, and excited-state intramolecular proton transfer, in the context of their applications for the detection of NTs. We also discuss the significance of NTs in human physiological functions, address the current challenges in designing fluorescence-based biosensors for the detection of NTs, and explore their future development.

## 1. Introduction

In biomedical research, understanding the complex functions of the brain and its signal transduction remains the greatest challenge to the scientific community. The nervous system is a complex intracellular communication system in which neurons are specialized cells that receive, process, and transmit signals throughout an organism. This process is commonly referred to as neurotransmission. Neurotransmission occurs through the release of specific biomolecules known as NTs [1]. These endogenous chemical messengers are crucial for the transmission, enhancement, and conversion of specific signals between the neurons and other cells. The fluctuating concentrations of NTs cause many mental and physical disorders, including Alzheimer’s disease, Parkinson’s disease, schizophrenia, Huntington’s disease, cardiac arrhythmias, depression, and anxiety. Several chemical messengers have been identified, making it essential to understand the role of NTs and quantify their levels for the early diagnosis of neurological disorders [2]. To make it convenient for readers, Table 1 illustrated some of the neurotransmitters and their recommended levels in practical use.

Biosensors can detect or quantify many of the analytes in biological systems. To date, several conventional methods have been used to quantify NTs, which include classical micro dialysis technique [3], high-pressure liquid chromatography (HPLC) [4], mass spectroscopy (MS) [5], capillary electrophoresis (CE) [6], magnetic resonance imaging [7], and electroencephalography [8]. Compared to other classical techniques, the micro dialysis is a gold-standard technique for detecting NTs, but it has poor temporal resolution. However, these classical techniques are time-consuming, require a significant sample volume, and are unsuitable for efficient detection [9]. Recently, biosensors, especially electrochemical-based techniques, have been used to solve the temporal resolution problem, which is improved considerably and highly specific and significant information is provided at the cellular level in a relatively fast manner. Biosensor-based electrochemical methods faced some problems related to lack of specificity to detect chemical species in the brain that have similar redox potentials and lack of detection of non-electroactive biomolecules. In recent decades, to overcome the above issues, fluorescence biosensor platforms emerged with advanced nanomaterials and biomaterials to develop the techniques for the in vitro and in vivo detection of NTs. However, sample-based biomarkers used in clinical diagnostics are currently insufficient and require a high degree of biosensing. Fluorescence biosensors are gaining significant attention in biosensing applications because of their simplicity, high sensitivity, selectivity, cost-efficiency, and rapid response.

Nanomaterials are promising building blocks for designing biosensors based on NTs. Nanomaterials, including nanoparticles (NPs), nanotubes (NT), nanoclusters (NCs), nanorods (NRs), nanocomposites (NC), and quantum dots (QDs) of metals, metal oxides, carbon, polymers, and enzyme-based materials, exhibit unique physical properties. These nanomaterials have been used to design sensors for the detection of NTs. The advent of new generations of engineered nanomaterials through methods such as functionalization and surface modification, as well as the development of various composites, has expanded the possibilities of achieving a highly sensitive, picogram-scale detection of NTs [10]. Various fluorescence mechanisms have been used for the detection of NTs, including the enhancement of emission through the passivation of NPs on the surface of the analyte that reduces the emission via the quenching effect of NPs and nonradiative Förster resonance energy transfer (FRET).

In the last few decades, fluorescent biosensors have made a great impact in the detection of biomolecules by means of biosensing and bioimaging. To date, there have been some reviews published regarding fluorescent biosensors for neurotransmitter detection. Leopold et al. summarized various fluorescence biosensors for neurotransmission and neuromodulation and their engineering aspects for designing fluorescence-based biosensors [11]. Rasheed et al. summarizes recent advances in the use of various nanomaterials and their application in the optical determination of dopamine [12]. Lu et al. summarizes relevant neurotransmitters and recent works related to aptamer biosensors for the detection of NTs. In this review, we deeply discuss the widespread view of various nanomaterials, their efficiency, and the mechanisms used in the construction of fluorescence-based biosensors for NTs detection. We discuss various NTs, their role in physiological functions, and the detection in the early stages of neurological disorders.

In addition, we explore the various fluorescence-based biosensors that use diverse nanomaterials, including metal clusters, graphene dots, and carbon dots (CDs), as well as biomaterials such as enzymes, aptamers, and genetically encoded (GE) proteins for the cellular-level detection of NTs. Finally, we discuss the major challenges for and perspectives on fluorescence biosensors. We believe that this review is pioneering to help to facilitate the design of smart sensing strategies using various materials based on nano and biological concerns, thereby promoting the successful development of fluorescence-based biosensors for detecting NTs and ultimately enhancing disease prognosis.

**Table 1:** Recommended level of neurotransmitters concentration in plasma.

S.no	NTs	Plasma	Ref.
1	Dopamine	0 to 30 pg/mL	[13]
	Epinephrine	0 to 140 pg/mL	[14]
2	Nor epinephrine	70 to 1700 pg/mL	[14]
3	Serotonin	50 to 200 ng/mL	[13]
4	Gamma-amino butyric Acid (GABA)	30 to 79 pmol/mL	[15]
5	Acetylcholine (ACh)	0.20 to 1.31 µmole/L	[13]
6	Glutamate	40 to 60 μM	[16]

## 2. Principle of Fluorescence-Based Biosensors

Fluorescence-based biosensors are widely used in various fields such as medicine, physiology, environmental studies, and pharmacology because of their simplicity, cost-effectiveness, and high sensitivity. Fluorescence is a phenomenon in which a substance absorbs light of a higher energy or shorter wavelength and emits light of a longer wavelength. The emitted light, which is short-lived and typically lasts for 10^−8^ to 10^−9^ s^−1^, is defined as fluorescence [17]. There is considerable interest in exploring the signaling mechanisms between target molecules and fluorescent molecules or dyes used as signal-reporting units to develop novel fluorescent biosensors for detecting supramolecules at the molecular level. The photon–physical mechanism at the molecular level and the principles of supramolecular interactions offers distinct advantages for developing novel and well-organized fluorescence sensors. Conventional mechanisms for molecule detection, including fluorescence resonance energy transfer (FRET), photon-induced electron transfer (PET), intramolecular charge transfer (ICT), excited-state intramolecular proton transfer (ESIPT), and dual- or triple-sensing mechanisms, have been developed. Here we discuss some of the mechanisms for reader convenience in the following sessions.

### 2.1. Fluorescence Resonance Energy Transfer (FRET)

**Figure 1:**
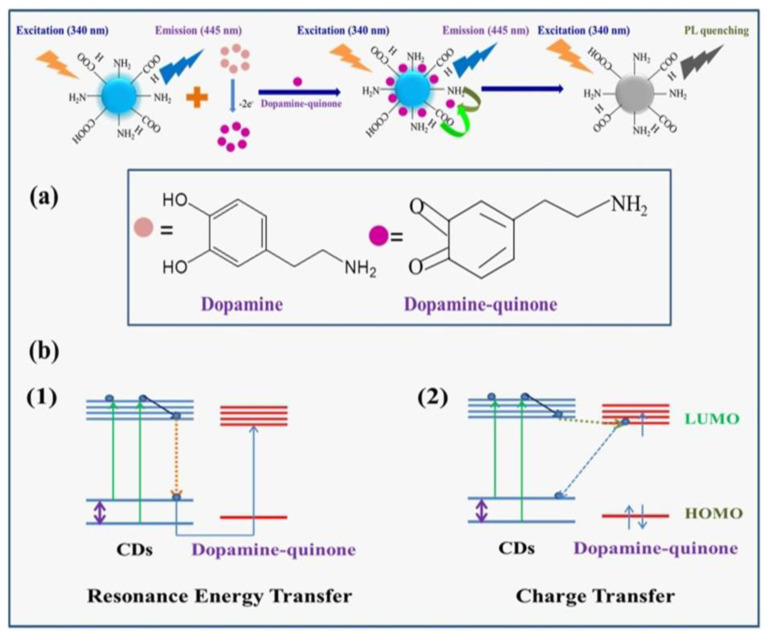
(**a**) Schematic representation of the FRET-based sensing platform of dopamine sensing as well as (**b**) diagrammatic representation of (**1**) electron-transfer process and (**2**) charge-transfer process from excited-state CDs to the ground state of dopamine–quinone. Reprinted from ref. [20]. Copyright (2020) with permission from Elsevier.

FRET is an important physical phenomenon in which energy transfer occurs between two molecules and was first described over 50 years ago; now, it is widely used in the detection of various biological systems and drug delivery. The basic principle of FRET is the mechanism associated with fluorescence and involves a nonradiative, long-range dipole–dipole coupling mechanism between the donor and acceptor. This fluorescence occurs when the emission spectrum of the donor partially overlaps with that of the acceptor. Upon excitation, electrons from the donor fluorophore transition from the ground state to the high-energy level of the acceptor fluorophore via FRET. A strong fluorescence signal is obtained from the probe when the quantum yield of the acceptor fluorophore exceeds that of the donor fluorophore. Conversely, a quenching mechanism is observed when the quantum yield of the acceptor fluorophore is lower than the donor fluorophore [18,19]. The quantum efficiency of the energy transfer in FRET is represented as the FRET efficiency, denoted as E, which is calculated using the Förster equation,

E = 1/[1 + (R/R_0_)^6^] (1)

where E is the efficiency, R is the distance between the donor and acceptor, and R_0_ is the Förster radius or distance at which the energy-transfer efficiency is 50%. R_0_ can be expressed in Angstrom by the following equation,

R_0_ = 8.79 × 10^−5^ × [n^−4^ × Q × κ^2^ × J(λ)] (2)

where n is the refractive index of the medium in the spectral overlap, Q is the quantum yield of the donor in the absence of the acceptor, and J(λ) is the spectral overlap. κ^2^ is the orientation factor of two dipoles. Among the several photophysical phenomena that have been explored for the fluorescence-sensing mechanism, the FRET-based system is very effective and accruable for sensing applications. Amit Kumar et al. fabricated fluorescent carbon dots from porcine pancreatic lipase (PPL) [20]. The emission intensity of carbon dots is effectively quenched by dopamine quinone complex via the fluorescence resonance energy transfer (FRET) process, which is illustrated in Figure 1. This FRET process only occurs when donors and acceptors that have appropriate energy levels from the visible to near-infrared region are within the Förster distance of <50 Å.

### 2.2. Photon-Induced Electron Transfer (PET)

**Figure 2:**
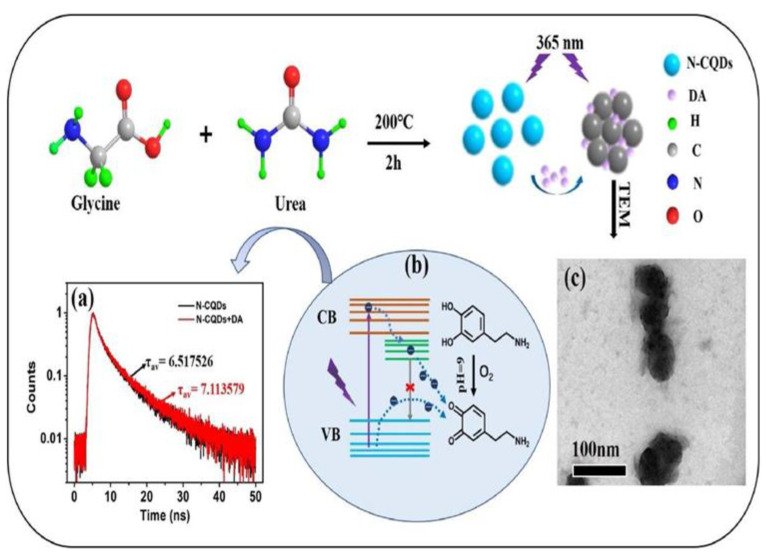
The mechanism of DA quenching the fluorescence of N-CQDs. (**a**) The PL lifetime decays of N-CQDs in the presence and absence of DA. (**b**) Schematic diagram of the transfer of electrons from N-CQDs to dopamine quinones. (**c**) The TEM of N-CQDs in the presence of DA. Reprinted from ref. [22]. Copyright (2020) with permission from Elsevier.

PET is a fundamental electron-transfer mechanism used to design efficient fluorescent probes. In the PET process, electrons move from the activating group to the fluorophore upon exposure to light. This process occurs when the electron in the highest occupied molecular orbital (HOMO) is transferred to the unoccupied HOMO of the excited-state fluorophore. Once fluorescence is induced, the electrons in the lowest occupied molecular orbital (LUMO) cannot return to their ground state [21]. Understanding the PET mechanism is crucial for designing the ‘‘off-on’’ or ‘‘on-off’’ fluorescent probes. When target analytes are recognized, the binding of the recognition or activating group to the target analyte can result in either fluorescence being turned on (‘‘off-on’’) because of PET being restricted or fluorescence being turned off (‘‘on-off’’) because of PET being activated. Wang et al. developed a detection system based on the PET process for the detection of DA, which is explained in Figure 2 [22]. This system is fabricated using carbon quantum dots doped with nitrogen atoms from glycine and urea by the facile solid heat method. Under alkali conditions, dopamine is oxidized to quinones by the electron transfer resulting in fluorescence quenching. In the PET process, the fluorescence enhancement process is more satisfactory than the quenching process, because the fluorescence enhancement process has higher selectivity due to the interaction of analytes to restore the fluorescence. The enhancement in fluorescence by quenching makes it easier to observe signals in dark backgrounds than bright backgrounds. However, the scope and application of the PET-based system is still limited. For example, in the case of cancer diagnosis and treatment, there is a demand to measure the cell polarity using a PET-based fluorescent probe.

### 2.3. Excited-State Intramolecular Proton Transfer (ESIPT)

**Figure 3:**
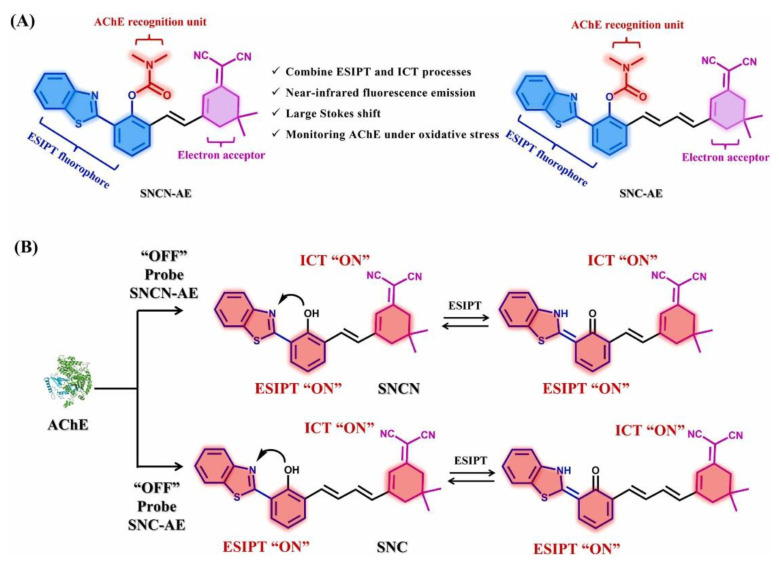
(**A**) Molecular structures of probe SNCN-AE and SNC-AE. (**B**) Sensing mechanism for detecting AChE. Reprinted from ref. [25]. Copyright (2023) with permission from Elsevier.

ESIPT is a process in which proton exchange occurs via the intramolecular hydrogen bonding between the proton donor and acceptor groups within a molecule. To improve the detection efficiency, the proton migrate between donor and acceptor atom is accompanied by large Stokes shifts. The first study of ESIPT for salicylic acid was reported by Weller in the 1950s [23]. The most common ESIPT fluorophores include 2-(20-hydroxyphenyl) benzimidazole, 2-(20-hydroxyphenyl) benzoxazole, and 2-(20-hydroxyphenyl) benzothiazole. Most ESIPT fluorophores have dual emission, large Stokes shift and high sensitivity to microenvironment. During photoexcitation, the molecules undergo a redistribution of electronic charge, increasing the acidity of the hydrogen bond donor group and the basicity of the hydrogen bond acceptor group within the E form [24]. Because of this rapid photoexcitation, ESIPT fluorophores are widely used as fluorescent probes, biosensing elements, and imaging agents in bioimaging techniques. Wei et al., designed a probe that combines ESIPT and ICT processes to make specific detection of AChE activity, which is shown in Figure 3 [25]. This enzyme activity interferes with the Ach metabolism and its over expression is related to many neurodegenerative diseases. It is believed that the fluorescent probe utilizing the ESIPT sensing mechanism will provide good photostability and perform with large Stokes shift.

### 2.4. Aggregation-Induced Emission (AIE)

**Figure 4:**
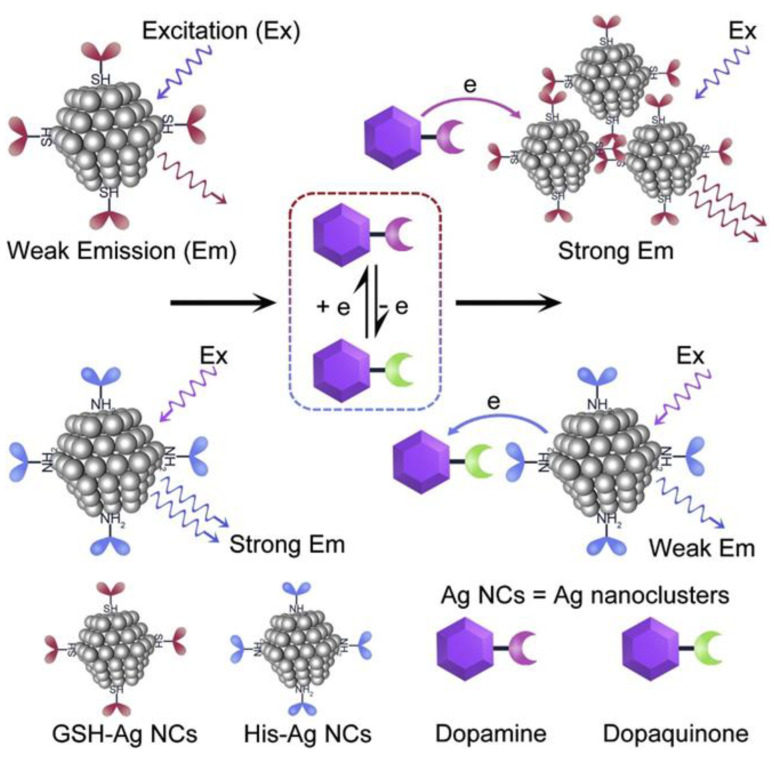
Sensing mechanism of GSH-DHLA-Ag nanoclusters and His-Ag nanoclusters for the ratio detection of DA. Reprinted from ref. [34]. Copyright (2020) with permission from Elsevier.

AIE has garnered significant attention in the scientific and research communities because of its broad applications in biomedicine. This phenomenon, discovered by Tang et al., involves a class of fluorescent molecules that exhibit high-emission efficiency when aggregated while showing weak or no fluorescence when dissolved [26]. The AIE fluorescent molecules emit weaker or non-emissive fluorescence in solution, but emit strongly in the aggregated state due to restriction of intramolecular motions. Common AIE fluorescent molecules such as 1-methyl-1,2,3,4,5-pentaphenylsilole (silole), tetraphenylethene, and anthracene derivatives have been successfully synthesized [27]. Materials with AIE properties have been extensively used in bioimaging [28], sensing [29] and therapeutics [30]. Beyond the fluorescent molecules so far used to develop AIE materials, a series of methods have been employed for the introduction of AIE molecules into the nanoparticles, which includes the reprecipitation method [31], amphilic molecule encapsulating method [32], silica-coating methods [33]. etc. Recently Ling et al., developed the ratio metric fluorescent biosensor for the detection of dopamine based on aggregated induced emission using ligand-templated silver nanoclusters [34]. Ag nanoclusters (Ag NCs) are prepared in two ways, using His, GSH and dihydrolipoic acid (DHPA). Ag NCs prepared with His exhibit blue fluorescence and those with and GSH and dihydrolipoic acid (DHPA) exhibit red color fluorescence. During the detection of DA, DA is oxidized to dopaquinone, an electron acceptor, blue fluorescence was quenched. In Ag NCs with GSH upon addition of DA, aggregation occurred resulted in fluorescence enhancement occurring, which is explained clearly in Figure 4. Fluorescent probe and sensor were developed by incorporating AI-induced material which makes it highly suitable for the sensing of biomolecules, drug molecules, monitoring food safety and environmental hazards.

### 2.5. Dual- or Triple-Sensing Mechanisms

**Figure 5:**
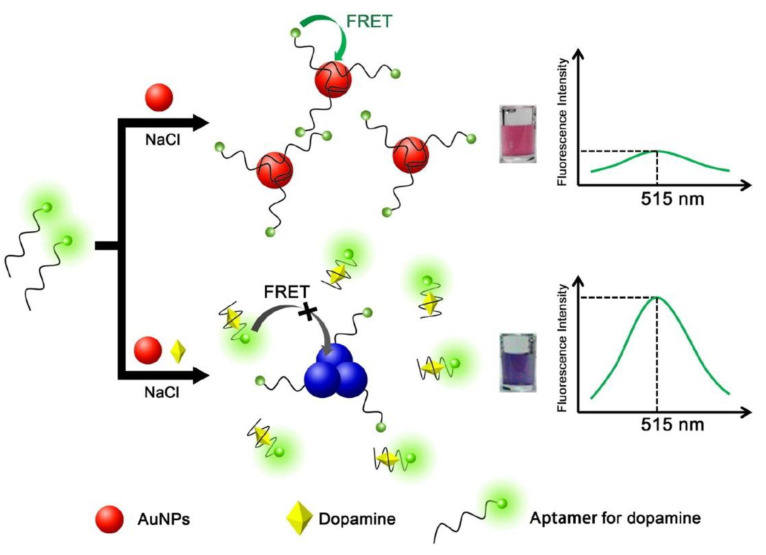
Mechanism for dopamine detection using FAM-labeled aptamers and AuNPs. Reprinted from ref. [36] Copyright (2016) with permission from Elsevier.

The scientific community has recently shown a substantial interest in the development of sensors based on dual- or triple-sensing mechanisms. Fluorescent sensors based on dual- or triple-sensing mechanisms produce diverse signals from fluorescent molecules. These mechanisms simultaneously sense multiple analytes with high selectivity and sensitivity [35]. Conventional single-sensing mechanisms may yield one or two types of fluorescent signals from the analyte, limiting their ability to provide multiple signals for the detection of interplaying biomolecules in complex living systems. However, dual-sensing mechanisms (e.g., FRET-ICT, FRET-PET, TBET-PET, ICT-PET, ESIPT-AIE, ESIPT-PET, ESIPT-FRET, and ESIPT-ICT) or triple-sensing mechanisms (PET-ICT-ESIPT and PET-ICT-FRET) can simultaneously sense multiple analytes. These mechanisms can be employed in the design of smart logic gates with multiple inputs and outputs. With the advantage of nanomaterials, several dual modes of sensors have been developed based on calorimetric and fluorescence biosensing methods. These types of sensors are more sensitive, accurate, and reliable. Zhang et al. developed a dual mode system for the detection of dopamine in human plasma. This dual system consists of AuNPs and fluorophore-labeled dopamine-binding aptamers [36]. This dual mode system, in colorimetric analysis worked based on the change in color AuNPs induced by dopamine and the fluorometric analysis is based on the fluorescence recovery induced by dopamine. The mechanism of this system is illustrated in Figure 5. This sensing system showed excellent detection of DA in complex biological samples. Compared with other fluorescence-sensing methods, the dual-mode sensing system includes simplicity, rapid analysis, very comprehensive qualitative and quantitative results, and robustness in complex biological samples.

## 3. Neurotransmitters and Their Importance

**Figure 6:**
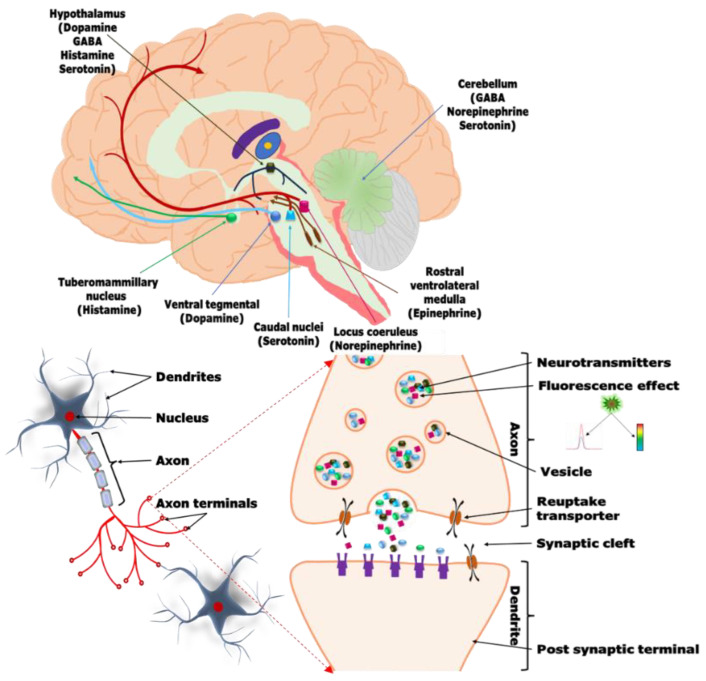
The Schematic diagram shows the basic mechanism of neurotransmission, neurotransmitters, and fluorescence sensing. Release of neurotransmitters from presynaptic neurons and individual biosensors are capable of detecting depending on the concentration and distribution of the probes by fluorescence signal intensity.

**Figure 7:**
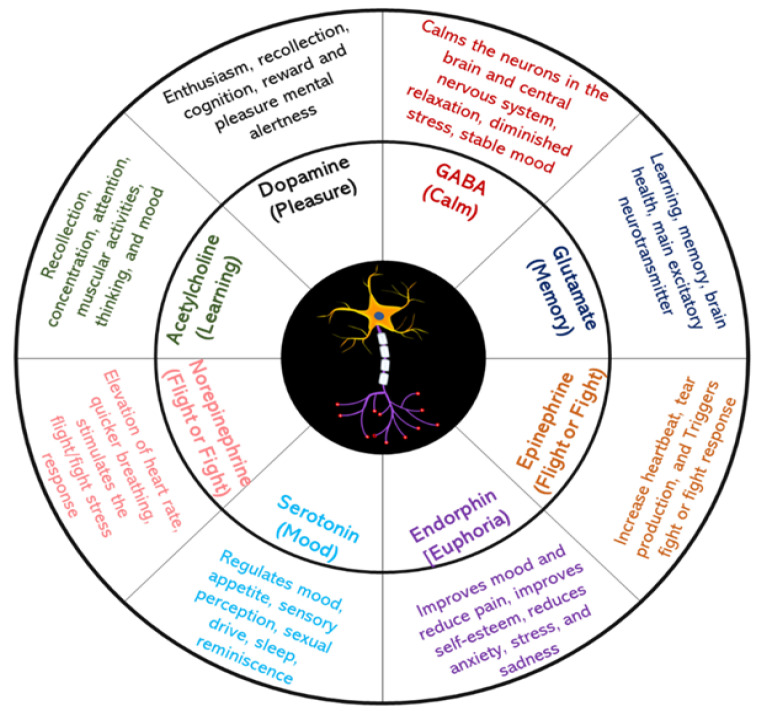
Illustration of major neurotransmitters and their functions.

NTs are small molecules that amplify, transmit, and convert signals in the cells of the central and peripheral nervous systems. Communication between two neurons happens in synaptic cleft which is the small gap between the synapses of neurons. From Figure 6 it is well described the schematic presentation how the electrical signals that are travelled along with axons and that are converted into chemical signal through the participation of neurotransmitters causing a specific response in the receiving neuron. Several NTs have been identified and these are either small amine molecules, amino acids, or neuropeptides. For the reader’s convenience, important neurotransmitters and their functions are clearly described in Figure 7. In the following section, we briefly discuss some of the neurotransmitters, their significance, and current detection strategies.

### 3.1. Dopamine (DA)

DA (3,4-dihydroxytyramine) is an NT that plays a vital role in the central nervous system as well as in the renal, cardiovascular, and hormonal systems [37]. DA is a type of catecholamine and belongs to phenethylamine families. Between 1957 and 1959, Kathleen Montagu and her colleagues at the Hans Weil-Malherbe lab at Runwell Hospital (England) and Arvin Carlsson and his colleagues at Lund University (Sweden) collectively found DA along with other catecholamine, hydroxytyramine, EP, and NP, and its function as a neurotransmitter in brain. The 3, 4-dihydroxytyramine, DA made up of a benzene ring with two hydroxyl side groups attached to one amine group via an ethyl group. Dopaminergic neurons that are present in the brain produce DA from tyrosine via the addition of hydroxyl group, that transforms it into L-DOPA and the subsequent removal of a carboxylic acid group from the ethyl side chain linked to the amine group results in DA [38]. DA binds to five subtype DA receptors, D1, D2, D3, D4, and D5, which belong to G-protein-coupled receptor family and initiate the signaling response for functions that are associated with the brain.

DA plays as important role in various physiological functions, including motor movement and motivation. Understanding various biological functions and mechanisms necessitates the measurement of DA level. Elevated levels of DA can result in cardiotoxicity, while low levels may indicate neuronal disorders such as Parkinson’s disease [39], Alzheimer’s disease [40], and depressive disorders [41]. Various analytical methods have been used to detect low levels of DA, including CE, rapid liquid chromatography-tandem mass spectrometry [42], conventional chromatographic techniques, and enzyme assays [43]. Additionally, various detection techniques such as ultraviolet [44], fluorescence detection [45], chemiluminescence [46], and electrochemical detection (ECD) [47] have been developed. These techniques are widely accepted as reliable; however, they have limitations, such as high costs, time-consuming procedures, labor-intensive processes, and dependence on specific instruments. Consequently, there is a need to develop a reliable technique for DA detection.

### 3.2. Epinephrine (EP)

EP or adrenaline [1-(3,4-dihydroxyphenyl)-2-methyloamino-ethanol)] is a sympathomimetic catecholamine belonging to members of the catecholamine group and is a vital NT in the central nervous system. It is also present in nervous tissues and body fluids as large cations. EP exhibits its pharmacological effects on its two adrenergic receptors, alpha and beta using G protein-linked second messenger system. Its affinity varies among receptors, greater affinity for beta receptors in small doses and selective affinity for alpha receptors in large doses. EP initiates the “flight or fight reaction” in response to situations such as violent events or intense emotions such as uncontrolled anger, fear, and other strong feelings. This response triggers the secretion of EP, resulting in increased blood pressure, sugar metabolism, heart rate, and muscle contractions [48]. EP is present in humans at the nanomolar (nM) level, and its quantification is significant in disease diagnosis assays. Even at low concentrations, EP plays a pivotal role in metabolic processes and has been associated with various significant illnesses such as Parkinson’s disease, Alzheimer’s disease, and neurological disorders. Furthermore, EP is crucial in various other metabolic processes, including the gastrointestinal innate immune system and nutrient absorption. It is also important in medical treatment, serving as a resuscitating agent following a heart attack and in managing bronchial asthma attacks. This property of EP is due to its ability to stimulate beta receptors, resulting in vasodilation at low doses (which increases blood flow) and vasoconstriction at higher doses [49].

Conventional methods for detecting EP include HPLC [50] and other fast detection techniques such as CE [51], electrochemical analysis [52] and optical spectroscopy [53]. While these conventional methods have been used, they may not always provide satisfactory detection limits and are often time-consuming with low selectivity. The biosensor-based detection systems provide alternatives to the techniques; however, only a few techniques are available for detecting EP. Detecting EP is challenging because of its rapid metabolism. With the expedited growth of nanobiotechnology, various nanomaterials, nanocomposites, and QDs are widely used in the construction of nano sensors. An electrochemical biosensor was employed to detect EP; however, it exhibited a limited detection range. The fluorometric methods are sensitive and selective, making them suitable for the rapid detection of EP at very low concentrations in human blood samples. Nevertheless, developing sensitive and selective methods for EP detection remains challenging because of the structural similarities between other NTs and various metabolites, including amino acids, as well as potential interferences from other drugs.

### 3.3. Norepinephrine (NE)

NE (noradrenaline) is also sympathomimetic catecholamine derived from l-tyrosine, an aromatic amino acid and primarily synthesized in the locus coeruleus (LC). NP differs from EP by structural variation by the absence of methyl group on the nitrogen atom. It is widely present in the central nervous system and specific regions of the cerebral cortex and peripheral organs in vertebrates. NE acts on both alpha- and beta-adrenergic receptors, in this sense while it acts on alpha-adrenergic receptors as a peripheral vasoconstrictor and act on beta-adrenergic receptors as an inotropic stimulator of the heart and dilator of coronary arteries. NE is an endogenous hormone secreted by noradrenergic neurons in the adrenal medulla of the central nervous system and plays a pivotal role in various physiological functions. These functions include memory [54], the regulation of sleep–wake or arousal states [55], response to stress, and the processing of sensory information [56].

The quantitative determination of NE compared with other catecholamine NTs is challenging because of their structural similarities and low concentrations in human samples. Given its highly complex nature, a highly selective and specific quantification method is imperative. Current conventional methods such as ultra-HPLC and micro dialysis have limitations when determining NE activity in its physiological state because of the low temporal resolution and complex sampling procedures [57]. The ECD methods, including fast-scan cyclic voltammetry, offer high temporal resolution in the millisecond range and exhibit sensitivity down to the nano-molar level. However, these methods often lack the specificity to distinguish NEP from other NTs. Compared with traditional analytical methods, fluorescence should be alternative methods due to its robustness, sensitivity, and good responsiveness to make real-time analysis to detect NE.

### 3.4. Serotonin

Serotonin, also known as 5-hydroxytryptamine (5-HT), is a monoamine that plays a significant role in mood regulation, behavior, cognition, and numerous neuro physiological processes. A severe drop in the level of serotonin will lead to Alzheimer’s diseases [58], and due to its mitogenic property it acts as cancer biomarker [59]. 5-hydroxyindole-3-acetic acid (5-HI-3-AA) is an important metabolite of serotonin and its abnormal level in urine showed important biomarker for neuroendocrine tumor [60]. It is essential to monitor the serotonin level in serum and 5-HI-3-AA level in urine to make early diagnosis of neurological and cancer-related diseases. Several classical techniques such as liquid chromatography–mass spectrometry (LC–MS) [61], CE [62] and enzyme-based immuno assay [63] were used to quantify the serotonin level. The analysis of serotonin in brain tissue was carried out by an extraction method using strong acids [64]. In the detection process, serotonin was subjected to a reaction with ninhydrin, resulting in a high fluorescence sensitivity compared with the native fluorescence. The fluorescence of this resulting product was eight times more intense than that of the native product under strongly acidic conditions [65]. Serotonin is rapidly released upon stimulation and secreted during exposure to NE, indicating that it is physiologically active and secreted by the gland. Serotonin can also be detected in biological fluids using ethanolic extraction, which enhances the fluorescence intensity without requiring time-consuming steps. However, this technique is unsuitable for repeated analysis because serotonin in the blood undergoes rapid oxidation in the presence of oxyhemoglobin. Recently, electrochemical methods were also used to detect serotonin and 5-HI-3-AA in biological samples, but they showed low selectivity due to the interference of other NTs that have close oxidative potentials. To overcome these issues, fluorescence biosensors must be simple, selective, and robust to detect serotonin at the molecular level.

### 3.5. Gamma-Aminobutyric Acid (GABA)

GABA, a selective modulator, is a crucial inhibitory NT in the mammalian central nervous system. GABA plays a significant role in regulating excitability and participates in melatonin synthesis [66]. Consequently, melatonin modulates the response of GABA in sleep and during reproductive functions [67]. GABA, a receptor modulator, regulates insomnia [68], epilepsy [69], premenstrual syndrome [70], and anxiety [71]. Several classical methods were employed to detect serotonin such as HPLC [72], HPLC with evaporative light scattering detector (ELSD) [73], micro dialysis [74], and functional magnetic resonance imaging (fMRI) [75]. A fluorescein-based imaging probe binds to the GABA receptors and emits fluorescence. However, in an aqueous environment, the probe adopts a folded conformation, resulting in the quenching of emission because of the intermolecular interactions [76]. Several amino acid-binding receptors, such as proteins, serve as potential sites for NT binding, with some proteins in this family binding to GABA. The high affinity of the transporter is valuable in the engineering of GABA biosensors. However, these methods are costly, require highly skilled personnel, and involve longer analysis time. Currently, biosensors using fluorescence-based approaches offer rapid response times, high sensitivity, and can detect GABA at low levels.

### 3.6. Acetylcholine (ACh)

ACh is an endogenous NT synthesized from choline and acetyl coenzyme A using the enzyme choline acetyltransferase. This NT was first synthesized by Adolf von Baeyer in 1867 and was recognized as biologically active in 1906. Notably, ACh plays an important role in human behaviors such as arousal, attention, sleep, emotion, memory, and learning. The abnormal levels of Ach are associated with many physiological disorders, such as Parkinson’s disease, Alzheimer’s disease, schizophrenia spectrum disorders, Huntington’s disease [77], bipolar disorder [78], and dementia [79]. Over the past two decades, considerable efforts have been made to detect abnormal levels of ACh to develop safe and effective pharmacological treatments. Several traditional methods are employed for the detection of ACh, including gas chromatography [80] and HPLC [81]. However, these methods necessitate complex sample pretreatment and the use of expensive instruments for sample separation and purification. To overcome these issues, enzyme-based biosensors have been developed to detect choline and ACh. Choline oxidase and acetylcholinesterase are generally used to convert choline and ACh to H_2_O_2_. The formed H_2_O_2_ can then be detected using fluorescent, electrochemical [82], and colorimetric methods. Among these detection techniques, the enzyme-based fluorescence sensors are advantageous owing to their rapid response, easy sample preparation, high sensitivity, and selective detection of analytes.

### 3.7. Glutamate

Glutamate is the most abundant free amino acid, recognized as primary excitatory neurotransmitters in the mammalian central nervous system. Glutamate is unique among other neurotransmitters because of its function as a neurotransmitter as well as playing an important role in intermediary metabolism and as an important building block in protein and peptide synthesis, including glutathione [83]. Glutamate activity is mediated by two classes of receptors include both ionotropic (i.e., the N-methyl-d-aspartic acid (NMDA), α-amino-3hydroxyl-5-methyl-4-isooxazole-proprionate (AMPA), and kainate subtypes) and metabotropic glutamate receptors (mGluRs), which have eight subtypes metabotropic glutamate receptor (mGluR) 1–8). As a potent neuroexcitatory amino acid, glutamate and its glutamate receptors are implicated in several neurological disorders, including head and spinal cord trauma [84], Huntington’s [85], Parkinson’s, and Alzheimer’s disease [86], and epilepsy [87]. Apart from neurological diseases, glutamate serves as a diagnostic biomarker for various cancers [88] and diabetic conditions [89]. It is essential to monitor glutamate concentration, otherwise many diseases that are associated with high blood levels of glutamate may go undetected. Several classical techniques have been developed for glutamate detection including HPLC [90], capillary electrophoresis [91], micro dialysis [92], and enzyme-linked immune sorbent assay [93]. But these have limitations such as being time consuming, requiring expensive instruments, and being labor intensive. During the past two decades, advances in nanotechnology and electrochemical and fluorescence-based biosensors have received greater attention to achieve point-of-care detection, because these techniques are cost-effective, reliable, fast, and need a small volume of samples.

## 4. Fluorescence-Based Biosensors for NTs

### 4.1. Nanomaterial-Based Biosensors

Nanomaterials and nanostructured composites hold immense promise in advancing biosensing technologies, paving the way for the development of point-of-care biosensing devices that are poised to revolutionize healthcare diagnostics and therapeutic practices shortly. We listed some of the nanomaterials and their linear range of detection of various NTs in Table 2. Nanomaterials with fluorescent properties are currently undergoing rapid development in biosensors and their applications in the healthcare industry. The subsequent sections delve into some of the fluorescent-based nanomaterials in detail.

#### 4.1.1. Metal Nanoclusters (M-NCs)

M-NCs, or ultra-small NPs, are typically composed of fewer atoms, exhibiting diameters ranging from the sub-nanometer scale to approximately 2 nm. Their unique properties are increasingly gaining significance in biosensing applications. M-NCs possess unique characteristics, such as biocompatibility, tunable fluorescence dependent on scaffolding, strong photoluminescence (PL), and good photostability, thereby complementing conventional fluorophores [94]. The PL of metal-doped NCs (M = Au, Ag, Cu, and Pd) has been successfully used as potential sensory material. M-NCs are relatively unstable because of their extremely small size, necessitating the use of stabilizing agents to enhance their stability. The stabilizing agent or template furnishes a binding unit on the surface, thereby augmenting the sensing capability of the probe. This template also enhanced the biocompatibility and luminescence properties of M-NCs. The commonly used templates are thiols [95], dendrimers [96], DNA oligonucleotides [97], peptides [98], and proteins [99].

Gold nanoclusters (AuNCs) are widely used in biosensing applications because of their numerous advantages, including low toxicity, strong fluorescence stability, tunability, and facile surface modification. AuNCs have gained recognition as potential fluorescent materials compared with other fluorescent materials because they exhibit strong optical and magnetic properties, discrete redox behavior, HOMO-LUMO transitions, and optical chirality. Liu et al. synthesized the amino pillar [5] arene-AuNCs nanocomposite (AP5-AuNCs) for the highly sensitive and selective detection of DA. In this study, AuNCs were synthesized at room temperature via a two-step method that used glutathione (GSH) as a stabilizing agent and 11-mercaptoundecanoicacid as the reducing agent. The AP5 was attached to the surface of the AuNCs via the classical 1-ethyl-3-(3-dimethyl aminopropyl) carbodiimide hydrochloride/N-hydroxy-succinimide (EDC/NHS) condensation reaction, forming the AP5-AuNCs nanocomposite. The specific molecular recognition of this composite is due to the specific molecular recognition capability of the AP5 molecule with DA molecule; the size of the AP5 cavity (diameter 5.6 Å, high 7.8 Å) and size of the DA (one-dimensional size 4.5 Å) are perfectly combined to form a stable complex, which increases the quenching efficiency. The synthesized nanocomposite showed a linear range of DA detection ranging from 5 to 700 nM, with an LOD of 1.5 nM. The proposed system is simple, rapid, and has a wide linear detection range, allowing for the direct detection of DA in human urine samples without interference from other coexisting substances [100].

Over the past few decades, there has been a growing fascination with the protein-templated fluorescent AuNCs in biosensing because of their excellent optical properties and good biocompatibility. A strong red-fluorescent nanocomposite was constructed using graphite-like carbon nitride nanosheets (g-C_3_N_4_NSs) and serum albumin-capped AuNCs for DA detection. DA quenched the red fluorescence of the synthesized nanocomposite via FRET. This composite exhibited strong red fluorescence, with an emission at 420 nm that diminished proportionally in response to the concentration of DA. The study of interferents with other neurotransmitters and metal ions were carried out using this complex. Among the metal ions, Pb^2+^ ions show quenching effect, maybe due to formation of complex g-C3N4 NSs-AuNCs, but author stated that relative error is less than 5%. NE showed a 30% interference quenching effect, due to structural similarities between DA and NP. NP get easily oxidized to quinone but extend of oxidation was less compared to DA The quenching ratio demonstrated a linear relationship in the range of 0.05–8 µmol L^−1^, and the LOD was low at 0.018 µmol L^−1^. Using this nanocomposite, reliable and acceptable results were obtained for DA detection in human serum and urine samples [101].

The AuNCs were fabricated using trypsin as a ligand, enabling the selective sensing of multiple analytes. Trypsin, an enzyme with a molecular weight of 23.3 kDa, belongs to the serine-protease family. Trypsin has an active functional group capable of effectively reducing Au^3+^ ions, while trypsin-AuNCs offer novel sensing applications for detecting multiple analytes. The fabricated trypsin-AuNCs exhibited a strong red emission peak at 665 nm when excited at 520 nm. Notably, the LODs for carbidopa, DA, Cu^2+^, Co^2+^, and Hg^2+^ ions were 6.5, 0.14, 5.2, 0.0078, and 0.005 nM, respectively. Trypsin-AuNCs demonstrated good precision and accuracy when applied to actual biofluid samples for the detection of carbidopa, DA, and metal ions (Cu^2+^, Co^2+^, and Hg^2+^ ions) in biofluid and water samples [102].

**Table 2:** List of nanomaterial-based biosensors.

Nano Materials	Target	LOD	Linear Range	Ref.
Quantum dots (thioglycolic acid capped)	DA	2.55 nM	46.7 nM~0.394 μM	[103]
Quantum dots (Ndoped graphene)	DA	3.3 nM	10~3000 nM	[104]
Quantum dots (InP/ZnS)	DA	5 nM	0~100 nM	[105]
Quantum dots (Graphene)	DA	22 nM	1~40 μM	[106]
Quantum dots (Graphene)	DA	90 nM	0.25~50 μM	[107]
Carbon dots (boronic acid and amino groups)	DA	0.1 pM	1 Pm~1 μM	[108]
Carbon dots (Sulphur doped)	DA	47 pM	0~20 μM	[109]
Carbon dots (Nitrogen doped)	DA	5.54 μM	3.3~500 μM	[110]
N-Quantum dots (Nitrogen doped)	DA	0.07 µM	1~200 μM	[111]
N-Carbon dots (Glutathione modified)	DA	1.01 nM	20 nM~10 μM	[112]
Gold NFs	DA	0.21 nM	0.8~300 nM	[113]
Graphene Oxide	DA	94 nM	0.25~20 μM	[45]
Quantum dots (CdTe)	EP	6.8 nM	10 nM~20 μM	[114]
Quantum dots (CuInS_2_ capped by L-Cys)	EP	3.6 nM	1 × 10^–8^~1 × 10^–4^ M	[115]
Copper NPs	EP	0.2 μM	3 × 10^–5^~5 × 10^–7^ M	[116]
Carbon NPs	EP	88 nM	0.1~50 μM	[117]
Carbon NTs	EP	50 nM	2.3 nM~9.4 μM	[118]
Gold NCs	EP	910 pM	10~100 μM	[119]
Fluorescence dye	EP	0.14 nM	1~120 nM	[49]
Quantum dots (molecularly imprinted polymers)	NEP	9 nM	0.08~20 μM	[120]
Quantum dots (3-mercapropionic acid-coated CdTe QDs)	NEP	2.1 nM	5 nM~10 μM	[114]
Quantum dots (thioglycolic acid functionalized cadmium telluride (CdTe))	NEP	0.3 μM	2.5~20.0 μM	[121]
Quantum dots (molecularly imprinted polymer CdTe@ SiO_2_)	NEP	8 nM	0.04~10 μM	[122]
Gold NCs	NEP	49 nM	0.5~40.0 μM	[123]
Carbon NPs	NEP	91 nM	0.1~50 μM	[117]
Silver NPs	NEP	5.59 μM	8.92 × 10^–3^~5.66 × 10^–5^ M	[124]
Quantum dots (Mn^2+^ doped ZnS)	5-HT	3.91 nM	0.28~2.8 μM	[125]
Gold NCs	5-HT	49 nM	0.2~50 μM	[126]
rGraphene oxide	5-HT	55 nM	NA	[127]
Quantum dots (NADP+-functionalized)	GABA	22 nM	NA	[128]
Carbon dots (silica functionalized)	GABA	6.46 μM	0~90 μM	[129]

Bovine serum albumin (BSA) is a protein that can be used as a ligand to stabilize M-NCs. Zhou et al. fabricated the bimetallic gold-silver NCs (Au-AgNCs) using BSA as a stabilizing agent in a one-pot process [130]. The fabricated NCs exhibited a significant enhancement in fluorescence accompanied by a redshift of the peak when DA was introduced to the reaction system. An interference study with other interfering molecules showed that catechol only provides some fluorescence, because there are some structural similarities between catechol and DA. The extent of the fluorescence enhancement was directly proportional to the concentration of DA in the range of 0.01–1.7 μM and 1.7–10 μM. The LOD was calculated to be 6.9 nmol·L^−1^. Govindaraju et al. fabricated the protein immobilized AuNCs with a high quantum yield (approximately 12%) using BSA as a stabilizing agent via a reflux hydrothermal method. These AuNCs were used to detect EP in the serum of patients with Alzheimer’s disease [119]. In this study, the fluorescence intensity increased upon adding different concentrations of EP to the fabricated probe. This phenomenon can be attributed to the occurrence of PET during the polymerization of EP and surface passivation traps resulting from the abundance of conjugated double bonds between the electron-donating and electron-accepting functional groups. Compared to other interferents, EP only forms self-polymerization due to the oxidation of the catechol moiety. This probe demonstrated a remarkable level of sensitivity in the detection of EP in serum, with a LOD of 910 pM.

Aparna et al. synthesized the blue-emissive copper NCs (CuNCs) for DA detection. In this research, BSA was used as a capping agent to stabilize the CuNCs. The study involved the fabrication of two systems of blue-emissive fluorescence, denoted as CuNC1 (in the presence of BSA) and CuNC2 (in the absence of BSA), to investigate their respective fluorescence responses to DA in the presence of H_2_O_2_. These systems showed different intensities; BSA CuNC1 exhibited a higher intensity than BSA CuNC2. The difference in the intensities was attributed to the activation of BSA in the presence of H_2_O_2_ in BSA CuNCs. The probe was tested for other biomolecules, except L-Dopa other biomolecules have no quenching effect. Structural similarities of L-Dopa showed quenching effect with the probe. The author studied interference of L-Dopa, after addition of analyte DA, more quenching effect was observed. A linear response to DA was observed within the concentration range of 0.1 to 0.6 nM. The LOD for BSA CuNC1 was 0.1637 pM, while for BSA CuNC2, it was 0.024 nM. This system was effectively applied to the sensitive detection of DA in actual human serum and urine samples. The recovery rate of DA in serum samples was within the range of 90–98.33%, and for urine samples, it was in the range of 89–96.66%. They also developed a paper-strip sensor using BSA CuNC1 as a cost-effective and selective platform for DA detection [131]. M-NCs exhibit excellent fluorescent properties, possess nanoscale dimensions, and are biocompatible with cells. M-NCs have been demonstrated to enhance biosensor performance with a low LOD.

#### 4.1.2. Carbon Dots (CDs)

CDs are zero-dimensional NPs derived from carbonic nanomaterials with sizes less than 10 nm. CDs have attracted considerable attention owing to their wide range of properties, such as optical properties and fluorescence emissions. They belong to a large family of fluorescent materials with unique characteristics, such as good biocompatibility, high chemical stability, low cytotoxicity, excellent photochemical stability, increased efficiency, good surface functionality, and excellent physicochemical properties. CDs, as emerging fluorescent nanomaterials, have shown tremendous potential in paving the way for new approaches to drug delivery [132], genetic application [133], bioimaging [134], and photodynamic therapy [135].

The fluorescence properties of CDs depend on their size, shape, doped functional groups, and degree of crystallinity. CDs can emit fluorescence ranging from blue to red (400–700 nm) by altering the reagents and synthesis procedures [136]. In contrast, carbon NPs exhibit white fluorescence visible to the naked eye, characterized by a relatively weak fluorescence quantum yield, which can be enhanced through the chemical modification and surface passivation of functional groups [137].

Wang et al. prepared water-soluble N-doped CDs (NCDs) for DA detection. The NCDs were prepared via a hydrothermal process using CA and urea. The prepared NCDs were passivated with L-GSH to enhance their emissive properties. An intense blue emission was observed at 440 nm when excited at 355 nm. This system exhibited the quenching of the GSH-NCDs concerning DA concentration, with a linear detection range from 20 nM to 19 µM, and the LOD was 1.01 nM. With other interfering molecules, no quenching effect was observed. Interestingly, even though AA and UA have similar electrochemical properties with DA, no quenching effect was shown. These GSH-NCDs have been successfully used for DA detection in biological fluids [112]. Recently, CDs and their doped structures have provided a promising platform for the development of fluorescent probes to sense and detect DA. Through this approach, high-quantum-yield NCDs were synthesized, providing reliable, cost-effective, and visually observable fluorescent probes for detecting DA in human serum fluids. CDs and NCDs synthesized via the hydrothermal method exhibited high quantum yields of 54.29% and 89.82%, respectively. The linear ranges for DA detection using the CDs and NCDs were approximately 3.3–500 mM and 3.3–400 mM. The LODs for the CDs and NCDs were approximately 5.54 mM and 5.12 mM, respectively. These CDs and doped nanostructures exhibited high selectivity, sensitivity, and rapid response, making them potential fluorescent probes for detecting DA in human fluid samples [110].

Liu et al. synthesized water-soluble CuInS2 ternary quantum dots (QDs) capped with mercaptopropionic acid. The resulting quantum dots (F-CuInS2 QDs) showed symmetric fluorescence emission centered at 736 nm in the NIR region. The phenylboronic acid ligand formed boronated esters with vicinal diols. Compared with other molecules, DA, which possesses a vicinal diol group, shows a quenching effect with above probe [138]. Currently, most CDs are biocompatible, highly water-soluble, and less toxic. Consequently, their application in sensor development is inevitable. Therefore, there is a strong need to assess the nanotoxicity of CDs and develop environmentally friendly methods to synthesize biocompatible CDs.

#### 4.1.3. Graphene QDs (GQDs)

GQDs, zero-dimensional graphitic materials from the graphene family with dimensions less than 100 nm, have gained significant interest in biosensing technology. GQDs possess unique properties such as chemical inertness, low cytotoxicity, and excellent biocompatibility compared with conventional QDs. These superior qualities have generated interest in using this material in catalytic reactions and biosensing, bioimaging, and optoelectronic devices, as well as drug-delivery systems [139]. A simple and convenient method was used to develop a fluorescent probe for detecting DA and ascorbic acid using GQDs [140]. In this study, the interaction between DA and GQDs formed the DA-GQDs complex attributed to the electrostatic interactions and hydrogen bonding. To create a turn-off mechanism for DA detection, they added Cu^2+^ into this system. The catechol moiety in DA formed a complex with Cu^2+^ and was oxidized to o-semiquinone, resulting in a substantial fluorescence quenching of GQDs. Under optimum conditions, an excellent linear response to DA was observed in the range of 0.5–120 µmol L^−1^, with an LOD of 0.16 µmol L^−1^. This complex system exhibited good accuracy and repeatability for detecting DA in actual samples. However, the proposed method suffered from some limitations, including the need for a long analysis time and a high LOD for DA.

Elemental doping is one strategy for enhancing fluorescence performance by modifying the surface defect structure. The doping of graphene quantum dots (GQDs) with heteroatoms has garnered significant attentions, as the interactions between the GQDs and doped atoms can tune the functional properties of the GQDs. Many studies on nitrogen-doped GQDs (N-GQDs), sulphur-doped GQDs (S-GQDs), nitrogen- and sulphur-doped GQDs (NS-GQDs) [111] and nitrogen- and phosphorous-doped GQDs (NP-GQDs) have been conducted because of the unique properties of the doped elements in the GQDs [141,142,143,144]. A label-free fluorescence method and visible-paper-based strips were developed for DA detection using the N-GQDs. The N-GQDs were synthesized using a hydrothermal method by the carbonization of CA with ammonia [111]. The proposed mechanism of this system involves the oxidation of DA in the presence of O_2_ under alkaline conditions, leading to the transfer of electrons between the photoexcited N-GQDs and DA-quinine. Under optimized conditions, a good linear relationship was observed for DA detection in the range of 1–200 µM. Among the biological molecules incudes amino acids, and metal ions tested, only amino acids showed some interference which may be due to the presence of active groups in the amino acid. These N-GQDs were also used to detect DA in human serum samples, yielding satisfactory results. Similar studies have investigated the influence of elemental doping in GQDs for DA sensing. In this context, N-, S-, and NS-GQDs were synthesized [104]. The results were intriguing to discuss. The N-GQDs synthesized using urea and diethylamine as the nitrogen source exhibited a higher quantum yield and sensitive fluorescence-quenching performance for DA than the other doped GQDs. This is attributed to the higher nitrogen content in the N-GQDs. The diethylamine-doped GQDs exhibited a higher quantum yield than those doped with urea. However, no quenching performance was observed for DA with the diethylamine-doped GQDs. The X-ray photoelectron spectroscopy results revealed that the presence of pyridinic N in the N-GQDs synthesized using urea as a nitrogen source promoted the fluorescence quenching of the N-GQDs in response to DA. The authors found that the pyridine N-atoms can form delocalized electrons by contributing an electron pair to the π system across the carbon atoms, thereby accelerating the quenching mechanism. This complex structure excludes the interfering effect of other biomolecules, resulting in excellent sensitivity and selectivity, with a linear concentration range of 10–7000 nM and an LOD of 3.3 nM. Hence, there is a strong need to develop GQDs with substantial potential for applications in medical diagnosis, bioimaging, and sensing.

### 4.2. Enzyme-Based Biosensors

Enzymes, which function as biocatalysts, facilitate numerous physiological processes through enzyme-catalyzed reactions. To construct an enzyme-based biosensor, enzymes must be available for specific biochemical reactions and must be stable under the operating conditions [145]. Several enzymes, including tyramine oxidase, horseradish peroxidase, laccase, tyrosinase, and polyphenol oxidase, have been used for the detection of NTs. Li et al. developed a hybrid fluorescent probe, CDs/tyrosinase (CDs/TYR), for DA detection. In this system, DA, the analyte, is oxidized by tyrosinase to produce DA chrome. This product is then efficiently quenched on CDs, exhibiting a PL-quenching mechanism. This probe is not only low-cost but also stable, sensitive, and highly efficient for DA detection. It has a detection range of 1.318 × 10^−8^ to 2.06 × 10^−8^ mol L^−1^. Notably, this fluorescent probe eliminates the need for enzyme immobilization and modification, and the result can be read immediately upon completion of the probe-sample incubation [146].

A bioprobe was fabricated using CDs and the TYR enzyme. The TYR enzyme was conjugated through amide condensation, which involved the carboxyl group of CDs and the primary amine group of TYR. This probe catalyzed the oxidation of the DA, leading to the quenching of the fluorescence of CDs owing to an efficient energy transfer mechanism from the excited CDs. This mechanism releases the product dopaquinone (DQ). A selectivity study of this probe with potential interfering substances including common DOPA analogues, amino acids, and common ions was conducted. Several DOPA analogues showed interference with DA even their concentration is 30 times lower than DA. Amino acids and common ions also exhibit a little influence on the detection of DA. The specific catalytic activity of the conjugated TYR makes this bio-probe efficient for DA detection, with a linearity between 0.1 and 6.0 μM [147]. In both studies mentioned above, there was no need to immobilize the enzyme on the nanomaterials. The conjugation of the enzyme was sufficient to create a cost-effective bioprobe for DA detection.

Another cost-effective bioprobe was fabricated using CDs and laccase for DA detection. CDs were synthesized from curcumin and dimethylformamide using a microwave-assisted irradiation method. The synthesized CDs were then functionalized with a silicon precursor, 3-(aminopropyl)-triethoxysilane. The probe was constructed by immobilizing the laccase on CDs. This bioprobe demonstrated a significant fluorescence quenching for DA within a linear range of 0–30 μM, with a LOD of 41.2 nM. The mechanism underlying this detection is as follows: when DA is added to the bioprobe, it undergoes an oxidation reaction facilitated by the laccase in the bioprobe. This reaction converts DA into DQ. Subsequently, PET occurs between the bioprobe and DQ, wherein the CDD-CDs act as electron donors and DQ acts as an electron acceptor, leading to the PL quenching of the bioprobe [129].

A biosensor for EP detection has been developed using low-temperature cofired ceramics technology. This miniature biosensor incorporates a semiconducting polymer (poly(2,6-di([2,2′-bithiophen]-5-yl)-4-(5-hexylthiophen-2-yl) pyridine)), which was immobilized with enzymes such as laccases and tyrosinases from the oxidoreductase family [49]. The sensor operates by oxidizing the substrate in the presence of an enzyme to form a substrate-enzyme complex. These complexes do not emit fluorescence; hence, inorganic dyes based on ferric ions are used to generate fluorescence. Under optimized conditions, this system offers a wide linear detection range of approximately 0.14–2.10 nM. The system has been successfully used to detect EP in pharmaceutical samples.

A facile enzyme-based approach was used to detect GABA using the CDs derived from cornstarch selectively. In this study, CDs were functionalized with 3-aminophenyl boronic acid and nicotinamide adenine dinucleotide phosphate (NADP^+^) via the EDC/NHS coupling reaction [148]. The mechanism of the system involves the use of GABase enzyme by the functionalized CDs to detect GABA through fluorescence quenching. This occurs through the electron transfer between the enzyme and substrate owing to the formation of a reduced form of NADPH. This system showed some quenching effect with DA compared with other interferent molecules; this may be due to presence of catechol group in DA. This system detected GABA in the linear range of 0–90 μM with a LOD of 6.46 μM. The researchers employed this system to detect GABA in cerebrospinal fluid (CSF) and obtained recoveries of approximately 93.2–101.5% and 96.4–104.6%. Overall, the enzyme-based fluorescence biosensors are rapid, highly sensitive, and selective; however, they cannot be used in a wide range of concentrations below the nanomolar range. It is essential to immobilize the enzymes, and the use of nontoxic nanomaterials is encouraged to construct efficient enzyme-based biosensors.

### 4.3. Aptamer-Based Fluorescence Biosensors

Aptamers, small DNA or RNA sequences, bind to targets with high affinity and selectivity. These aptamers are selected in vitro through a process called systematic evolution of ligands by exponential enrichment [149]. Recently, aptamers have emerged as versatile alternatives to antibodies and other biomimetic receptors in the construction of biosensing devices and various biomedical applications. Aptamers are more stable than antibodies and economically viable because they are chemically synthesized as stranded oligonucleotides. Traditionally, molecular beacon aptamers (MBAs) have been used to engineer aptamers that exhibit FRET responses to specific biomarkers. The MBA, containing a donor fluorophore (fluorescein) and an acceptor fluorophore (quencher molecule), is attached to each end of the hairpin-loop oligonucleotide [150].

Various quencher molecules, such as AuNPs, QDs, graphene oxide (GO), and carbon nanotubes, have been used as fluorescent quenchers to detect different targets that bind to specific molecules. Teniou et al. developed an aptamer for the rapid detection of DA using GO as a quencher [151]. This sensor is based on the FRET mechanism, wherein GO acts as a fluorescence donor, and the carboxyfluorescein (FAM)-labeled aptamer is a quencher molecule. As a quencher molecule, the FAM-aptamer attaches to the surface of the donor GO through π–π stacking interactions between the nucleotide bases and carbon network. This leads to weak FRET and the quenching of the FAM fluorescence. When the target DA binds, the FAM-aptamer undergoes conformational changes, resulting in fluorescence recovery. This sensor showed minor responses in the presence of other interferences and detected DA concentrations linearly between 3 and 1680 nM, with an LOD of 0.031 nM and a limit of quantification of 0.1 nM. Wang et al. developed an aptamer-based sensor for DA detection, which operates on the DA-triggered Exonuclease III- and SYBR Green I-assisted template DNA using a cyclic amplification technique [152]. The template DNA, SYBR Green I, consists of two regions: one complementary to the complementary strand (cDNA), and the other complementary to the aptamer. In the presence of various concentrations of DA, DA triggers the Exonuclease III restriction digestion automatically to release SYBR Green I, leading to a significant decrease in fluorescence signal. This method achieved the linear detection of DA concentration in the range of 1.0 × 10^−10^ to 10.0 × 10^−9^ M, with an LOD of 8.0 × 10^−11^ M. This type of aptamer-based biosensor has been explored to study the metabolism and distribution of NTs and for the early diagnosis of abnormal NTs. Here we listed some of the aptamer and enzyme-based fluorescence biosensors for the detection of NTs in the Table 3.

Early studies on aptamer-based sensors for DA detection were hindered by a lack of fundamental understanding of aptamer binding and folding, which hampered the design of aptamer-based biosensors. To address this issue, a fast and simple aptamer-based fluorescent probe was developed using negatively charged single-walled carbon nano horns (SWCNHs), which acted as quenchers for DA detection [153]. DA-aptamers were labeled with 5-FAM. Because of the π–π interactions, the FAM-modified aptamers could be absorbed onto the surface of SWCNHs, decreasing the fluorescence intensity in the absence of DA. However, in the presence of DA, the DA interacts with the FAM-DNA aptamer to form a G-quadruplex, recovering the fluorescence intensity. This system enabled a linear range of DA detection from 0.02–2.20 mM with an LOD of approximately 5 μM. This innovative sensor was used to detect DA in serum samples.

Extensive research on the development of aptamer-based biosensors for the detection of NTs has been conducted. However, there has been limited research on fluorescence-based biosensors. The aptamer-based fluorescence biosensors have numerous potential impacts on the early diagnosis of diseases related to abnormal levels of NTs. However, most studies have only focused on a few NTs. Further research is needed for the other NTs, extending beyond clinical samples to potential samples for clinical development.

**Table 3:** List of aptamers and enzyme-based fluorescence biosensor for neurotransmitter detection.

S. No	Biosensor	Analyte	Fluorescence Probe	Nanomaterials	Sample	LOD	Linear Range	Ref.
1	Aptamer	DA	SYBR Green I	-	Mouse brain Tissue	8 × 10^−5^ µM	10^−4^~10^−3^ μM	[152]
2	Aptamer	DA	MoS2 Quantum dots as fluorophore	Quantum dots (Molybdenum disulphide)	Human serum	45 pM	0.1~1000 nM	[154]
3	Aptamer	DA	Ru complex	Quantum dots (RU Cadmium-Tellurim)	Fetal bovine serum	19 nM	0.03~0.21 μM	[155]
4	Aptamer	DA	Carboxy fluorescein (FAM)	Graphene oxide	Human serum	0.031 nM	3~1680 nM	[151]
5	Aptamer	DA	Carboxy fluorescein (FAM)	Graphene oxide	Human plasma and serum	1.0 nmol/L	-	[156]
6	Aptamer	DA	5-carboxy-fluorescein (FAM)	SWCNs	Human serum	5 μM	0.02~2.20 mM	[153]
7	Enzyme	DA	Adenosine monophosphate (AMP) and Cu^2+^	Polymer dots (Pdots@AMP-Cu)	Human serum	4 μM	10~400 μM	[157]
8	Enzyme	GABA	GABase enzyme	Carbon dots (corn juice)	Human serum	95.09 nM.	0–20 µM	[148]

### 4.4. GE Fluorescence Biosensors

The GE biosensors, which are genetically modified cells incorporating a chimeric reporter protein into their biochemical circuit [158], offer a wide range of detection capabilities. These biosensors enable the rapid, specific, and sensitive detection of analytes under physiological conditions, providing real-time analysis that outperforms other detection methods. They have become a versatile tool for imaging neuronal activity, especially for monitoring the release of NTs [159]. Some of the genetically encoded biosensors for neurotransmitter detection are listed in the Table 4. The chimeric reporter protein recognizes signals from the intracellular and extracellular fluctuations, converting them into measurable signals that reflect the optical changes within and outside living cells. The construction of a GE biosensor involves using a fluorescent protein to monitor the intracellular biochemical fluctuations [160]. The NT receptor, a crucial target-binding module for constructing the GE biosensors, is present in many of the approximately 800 NT receptors found in mammalian cells. A significant number of these receptors belong to G-protein-coupled receptors (GPCRs) [161], while a smaller family of NT receptors is associated with periplasmic-binding proteins [162].

**Figure 8:**
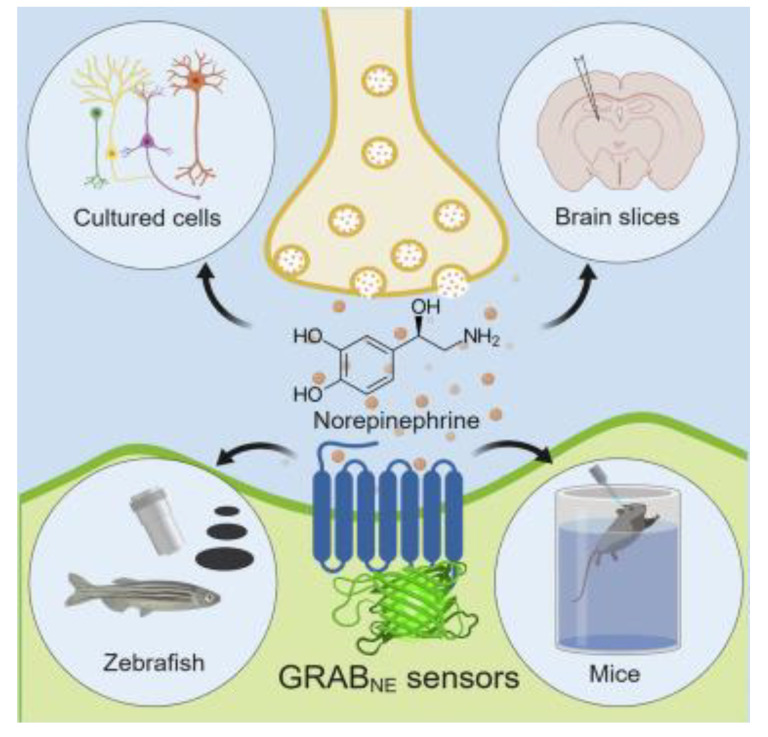
GE biosensors. Reprinted from ref. [168]. Copyright (2019) with permission from Elsevier.

Recently, the development of circularly permuted green fluorescent protein (cpGFP)-based sensors, such as the dLight, RdLight, and GPCR activation-based (GRAB_DA_) series, have been reported. These sensitive GE biosensors developed using cpGFP detect the DA concentration by binding with DA and inducing conformational changes in the fluorescence intensity of cpGFP. This is achieved by introducing the human DA D1 (DRD1), D2 (DRD2), and D4 receptors (DRD4) into the cpGFP module from the GE calcium indicator GCaMP6 [163]. The GRAB_DA_ sensors were developed by engineering the sensitive cpEGFP for a selective human DA receptor. The developed GE sensor was direct, rapid, sensitive, and performed cell-specific DA detection [169]. In this study, researchers developed two GRAB_DA_ sensor series: GRAB_DA1m_ (abbreviated to DA1m), with medium apparent affinity to DA (EC_50_ was approximately 130 nM), and GRAB_DA1h_ (abbreviated to DA1h), with high apparent affinity to DA (EC_50_ was approximately 10 nM). The developed GE biosensor demonstrated real-time sensitive detection of DA in the acute brain slices of mice and enabled detection in various animals, including fish, flies, zebrafish, and drosophila, as well as in cells and brain slice. Marvin et al. developed single-wavelength glutamate GE sensor, iGluSnFR, an optimized fluorescent probe for the detection of glutamate neurotransmission. This single-wavelength glutamate sensor was constructed from *E. coli* GltI and circularly permutated (cp) GFP. The developed iGluSnFR was bright and photostable, with 4.5 (∆F/F) max in vitro, under both one- and two-photon illumination, and in vivo it showed specific and fast sensitive detection of glutamate [170]. Marvin et al. applied the design and principles of glutamate receptor iGluSnFR for developing a biosensor for the detection of GABA [168]. The GABA-sensing fluorescence reporter (iGABASnFR) variants were developed from structure-guided mutagenesis and library screening of protein derived from an unsequenced *Pseudomonas* fluorescens strain. This iGABASnFR sensor is genetically encoded, detects in acute brain slices and produces readily detectable fluorescence increases in vivo in mice and zebrafish. By the continuation of this research, these researchers developed novel optical GABA sensor iGABASnFR2, an improved version of iGABASnFR [171]. This biosensor monitored extracellular GABA in hippocampal slices using patch-clamp GABA sniffer and showed periodic epileptiform discharges are preceded by transient, region-wide waves of extracellular GABA. A GE biosensor for NEP was developed from the family of GRABNE sensors, which is illustrated in Figure 8. These sensors exhibited a 230% peak ΔF/F_0_ response to NE, high specificity, sub-second kinetics, good photostability, and sensitive detection from nanomolar-to-micromolar concentrations [166]. The GRABNE sensors detect the release of NE from neurons of the LC, which responds to the electrical stimulation in mice and zebrafish, and report looming-induced NE release with single-cell resolution.

Nakamoto et al. developed a new red-fluorescent GE GPCR-activation reporter, termed ‘R-GenGAR-DA’, for the selective detection of DA in the presence of NE [165]. This system includes R-GenGAR-DA1.1, which brightens following DA stimulation, and R-GenGAR-DA1.2, a dimmed variant. These two variants were constructed by replacing the third intracellular loop of the human DA receptor D1 (DRD1) with a circular permutated red fluorescent protein (cpmApple), followed by the screening of mutants within the linkers between DRD1 and cpmApple. Among the two variants, R-GenGAR-DA1.2 demonstrated a reasonable dynamic range (ΔF/F_0_ = −43%), DA affinity (EC_50_ = 0.92 µM), and high selectivity for DA over NE (66-fold) in the HeLa cells. When combined with the green-NE biosensor GRABNE1m for dual-color fluorescence live imaging, the R-GenGAR-DA1.2 showed high selectivity for NE over DA (>350-fold) in the HeLa cells and hippocampal neurons. This GE biosensor represents the first step towards the multiple imaging of NTs to understand the role of DA and NE in normal and abnormal brain functions. In human physiology, the major cholinergic transmission was regulated by the neurotransmitters Ach. Jing et al. developed a family of G-protein-coupled receptor activation-based GE sensors for the in vivo and in vitro detection of ACh signals [173]. The developed GE sensors are validated in vitro using cultured cortical neurons, tissue slice from multiple brain regions and peripheral organs, and HEK293T cells, and in vivo using olfactory system of living drosophila and visual cortex of awake-behaving mice. The GACh sensors had sensitivity with an apparent affinity of 1µM specificity, a signal-to-noise ratio of about 14, kinetics of about τon/off ≈ 200–800 ms, and good photostability (≥1–4 h). This sensor is more precise and convenient to measure Ach signals in real sample analysis. 5-HT is an important monoamine NT that modulates physiological processes in the brain. Wan et al. developed a GE biosensor that encodes a GE GRAB and 5-HT sensor. The newly developed GRAB5-HT sensor exhibits a high affinity for 5-HT (EC_50_ was approximately 22 nM), relatively fast kinetics (τon was approximately 70 ms), high selectivity and sensitivity, and high spatial resolution. Additionally, it is well-suited for the direct detection of physiologically relevant endogenous 5-HT release [172].

GE biosensors have emerged as promising tools for identifying biological molecules in living systems. They are cost-effective, reliable, fast, and capable of real-time analysis. The GE biosensor excels in detecting physiological pathways with a high spatiotemporal resolution, studying the interactions between organelles and their biomolecules, and measuring the electrical responses to external stimuli. The GE-based biosensor, which is constructed using various cells and gene-editing tools, is effective in sensing technology. They validate various physiological functions and their abnormalities in living systems, making them invaluable tools in the field.

## 5. Conclusion and Future Perspective

NTs mediate synaptic transmission within the central and peripheral nervous systems. These endogenous chemical messengers carry signals, transmitting information from one nerve cell to another or different cell types. This neurochemical group comprises various molecules, such as amines, amino acids, and soluble gases. It is involved in many physiological functions, including memory, stress, sleep regulation, arousal, learning, movement control, and cognitive processes. Minor alterations in the concentrations of these NTs can cause abnormalities in physiological conditions, such as neurodegenerative disorders and cardiological issues. Therefore, a selective, sensitive, and rapid method is required to detect NTs at picomolar concentrations. Conventional methods require substantial human labor, involve long sample processing, and incur significant expenses. To address these challenges, researchers have developed novel techniques using smart nanomaterials, including CDs, GQDs, and biological molecules, which are cost-effective alternatives. Using nanomaterials such as QDs, M-NCs, and CDs not only enhances the sensitivity and selectivity of biosensors but also provides the real-time analysis of NTs, enabling rapid detection.

Over the past few decades, as a breakthrough in the field of nanotechnology, various nano-based materials for the construction of biosensors have emerged and been developed. There is increasing demand for biosensors in the health-care industry to monitor biomolecules, especially after COVID-19’s evolutions worldwide. The global biosensor market size was valued at USD 25.5 billion in 2021 and it is posed to grow from USD 27.41 billion in 2022 to USD 48.89 billion in 2030, with a growing CAGR of 7.5% in the forecast period of (2023–2030) [174]. Nano biosensors consist of nanomaterials that have dimensions ranging from 1 to 100 nanometers with biocompatibility and size tunability, making them efficient and accurate biosensors for treating life-threating diseases. In commercial aspects, nanomaterials such as quantum dots from carbon and graphene showed a steady and strong upward trend in recent years. It is anticipated that remarkable annual growth was observed for the carbon dots market from 2023 to 2030. Its application includes biomedicine, nano sensors, and optronic devices [175]. The technology applied for biosensors provides lab-level accurate and reproducible results anywhere in the world. But commercialization creates a challenge due to the need for the high initial costs from R&D and the slow rate of product commercialization and periods taken for approval that are anticipated to hinder the market revenue growth.

Nanomaterials with size, compatibility, tunability may clearly exhibit various roles in the development of the sensitive and selective detection of biomolecules. The current research scenario showed much attention to fluorescence-based biosensor for its easy operation, high sensitivity, and use of biocompatible materials. This review we discussed the various mechanism of fluorescence-based biosensors and their detection of neurotransmitters. Finally, we focus on the literature of nanomaterials and biomaterials including enzymes, aptamers, genetically encoded materials for the construction of fluorescence-based biosensors were discussed. This review provides an overview of various NTs and their physiological functions while exploring strategies and materials, particularly the fluorescence-based sensing materials, used to construct precise biosensors. The construction of fluorescent biosensors has addressed numerous challenges encountered in their application for medical diagnosis. The prospects for fluorescence-based biosensors are mainly focused on achieving specific and selective detection while ensuring cost-effectiveness. Presently, we are in the fifth generation of hospital on-chip generation, and the development of precise techniques is crucial for establishing a rapid and reliable method for the detection of NTs. With the emergence of artificial intelligence and cloud-based data, fluorescence-based biosensors have been upgraded for smart and remote accessibility, expanding their utility beyond the detection of analytes to encompass advanced drug delivery systems. Another challenge encountered in the development of biosensors is ensuring cost-effectiveness. When selecting materials for biosensor development, it is essential to ensure that they are biocompatible, eco-friendly, and relatively affordable. Along with innovation in technologies and theoretical studies to predict their structures, surface properties to make the ecofriendly synthesis of highly efficient nanomaterials will be achieved.

In summary, for the future generation of biosensors, especially in the context of sixth-generation biosensors for surgery on chips, the biosensor must not only enable rapid, sensitive, and selective detection but also be highly integrated, cost-effective, and capable of meeting all market demands. This review has discussed the significance of fluorescent biosensors in the detection of medically important NTs and explores the array of nanomaterials and biological materials available for designing high-demand fluorescent-based nano biosensors.

## Figures and Tables

**Table 4:** List of genetically encoded fluorescence biosensor for neurotransmitters detection.

Sensor Code	Analyte	EC_50_	ΔF/F0 (%)	Ref.
dLight1.1	DA	330 ± 30 nM	230 ± 9	[163]
dLight1.2	DA	770 ± 10 nM	340 ± 20	
dLight1.3a	DA	2300 ± 20 nM	660 ± 30	
dLight1.3b	DA	1680 ± 10 nM	930 ± 30	
dLight1.4	DA	4.1 ± 0.2 nM	170 ± 10	
dLight1.5	DA	110 ± 10 nM	180 ± 10	
R-GenGAR-DA	DA	0.7 µM	−50	[164]
R-GenGAR-DA	DA	0.92 µM	−43	[165]
GRABNE1h	NEP	83 nM	130	[166]
GRABNE1m	NEP	930 nM	230	
iSeroSnFR	Serotonin	10 mM	87 ± 20	[167]
iGABASnFR	GABA	240 µM		[168]
GRAB_DA1m_	DA	130 nM	90	[169]
IGluSnFR	Glutamate	4 µM	4.5	[170]
iGABASnFR2	GABA	1.1 µM	0.074 ± 0.006	[171]
GRAB 5HT	Serotonin	22 nM	280	[172]
GAch 2.0	ACh	2 µM	100.65 ± 7.61	[173]

## Data Availability

Not applicable.

## Abbreviations

Neurotransmitters	NTs
High-pressure liquid chromatography	HPLC
Mass Spectroscopy	MS
Capillary Electrophoresis	CE
Nanoparticles	NPs
Nanotubes	NT
Nanoclusters	NCs
Nanorods	NRs
Nanocomposites	NC
Quantum dots	QDs
carbon dots	CDs
Förster resonance energy transfer	FRET
Genetically encoded	GE
Photon-induced electron transfer	PET
Intramolecular charge transfer	ICT
Excited-state intramolecular proton transfer	ESIPT
Silver nanoclusters	Ag NCs
Glutathione	GSH
Dihydrolipoic acid	DHLA
Fluorescence resonance energy transfer–intramolecular charge transfer	FRET-ICT
Fluorescence resonance energy transfer–photo-induced electron transfer	FRET-PET
Through bond energy transfer–photo-induced electron transfer	TBET-PET
Intramolecular charge transfer–photo-induced electron transfer	ICT-PET
Excited-state intramolecular proton transfer–aggregated induced emission	ESIPT-AIE
Excited-state intramolecular proton transfer –photo-induced electron transfer	ESIPT-PET
Excited-state intramolecular proton transfer–fluorescence resonance energy transfer	ESIPT-FRET
Excited-state intramolecular proton transfer–intramolecular charge transfer	ESIPT-ICT
Photo-induced electron transfer–intramolecular charge transfer–excited-state intramolecular proton transfer	PET-ICT-ESIPT
Photo-induced electron transfer–intramolecular charge transfer–fluorescence resonance energy transfer	PET-ICT-FRET
Dopamine	DA
Epinephrine	EP
Norepinephrine	NP
Electrochemical detection	ECD
5-hydroxytryptamine	5-HT
5-hydroxyindole-3-acetic acid	5-HI-3-AA
Gamma-aminobutyric acid	GABA
Evaporative light scattering detector	ELSD
Acetylcholine	Ach
N-methyl-d-aspartic acid	NMDA
α-amino-3hydroxyl-5-methyl-4-isoxazole-propionate	AMPA
Metabotropic glutamate receptors	mGluRs
Metal nanoclusters	M-NCs
Photoluminescence	PL
Gold nanoclusters	AuNCs
1-ethyl-3-(3-dimethyl aminopropyl) carbodiimide hydrochloride/N-hydroxy-succinimide	EDC/NHS
Graphite-like carbon nitride nanosheets	g-C3N4NSs
Bovine serum albumin	BSA
Gold–silver NCs	Au-AgNCs
Copper NCs	CuNCs
Carbon dots	CDs
Ascorbic acid	AA
Uric acid	UA
N-doped CDs	NCDs
Limit of detection	LOD
N- and B-co-doped CQDs	NB-CQDs
Graphene QDs	GQDs
Nitrogen-doped GQDs	N-GQDs
Sulphur-doped GQDs	S-GQDs
Nitrogen- and sulphur-doped GQDs	NS-GQDs
Nitrogen- and phosphorous-doped GQDs	NP-GQDs
CDs/tyrosinase	CDs/TYR
Dopaquinone	DQ
Cerebrospinal fluid	CSF
Molecular beacon aptamers	MBAs
Complementary strand DNA	cDNA
Single-walled carbon nano horns	SWCNHs
G-protein-coupled receptors	GPCRs
